# Chylothorax after pulmonary resection and lymph node dissection for primary lung cancer; retrospective observational study

**DOI:** 10.1186/s13019-022-01758-1

**Published:** 2022-01-22

**Authors:** Yoshiyuki Yasuura, Hayato Konno, Takamitsu Hayakawa, Yukihiro Terada, Kiyomichi Mizuno, Hiroyuki Kayata, Hideaki Kojima, Tetsuya Mizuno, Mitsuhiro Isaka, Yasuhisa Ohde

**Affiliations:** Division of Thoracic Surgery, Shizuoka Cancer Center, 1007 Shimonagakubo, Nagaizumi-cho, Sunto-gun, Shizuoka, Japan

**Keywords:** Chylothorax, Lymph node dissection, Primary lung cancer, Pulmonary resection

## Abstract

**Background:**

Pulmonary resection with mediastinal lymph node dissection for treating primary lung cancer could sometimes causes chylothorax as a postoperative complication. This study examined the validity of treatments for chylothorax in our hospital.

**Methods:**

We evaluated 2019 patients who underwent lobectomy, bilobectomy, or pneumonectomy with mediastinal lymph node dissection for primary lung cancer at Shizuoka Cancer Center Hospital, Shizuoka, Japan, between September 2002 and March 2018. The diagnostic criteria for postoperative chylothorax were that the drainage from the pleural drain was evidently white and turbid, or the pleural effusion contained a triglyceride level of > 110 mg/dL. The clinical courses and treatments were retrospectively reviewed.

**Results:**

Postoperative chylothorax occurred in 37 patients (1.8%), 20 men and 17 women, with a median age of 70 years (33–80). A low-fat diet was instituted to all patients; 35 cases improved with conservative treatment, and 2 cases required reoperation. Nine cases had a drainage volume ≥ 500 mL one day following the low-fat diet commencement, which was resolved with conservative treatment and decreased drainage was observed on the third day of treatment in seven of those cases. Two cases with excessive drainage of ≥ 1000 mL in one day and systemic symptoms associated with chyle loss needed surgery.

**Conclusions:**

Even when the daily drainage volume exceeds 500 mL following a low-fat diet, there were many cases that could be cured conservatively. The indication for surgery needs to be carefully considered.

## Background

We sometimes experience chylothorax as a postoperative complication when performing lung resection with mediastinal lymph node dissection for primary lung cancer. It is caused by a thoracic duct injury and is characterized by the presence of chyle, composed of abundant triglycerides and chylomicrons, in the thoracic cavity [1]. Conservative treatment is successful in cases with minor thoracic duct damage. If conservative treatment is ineffective, the treatment strategy is gradually altered and eventually reoperation is considered. Chymic drainage of > 450 mL [2] or 500 mL [3, 4] per day is reportedly an indicator for reoperation; however, this treatment is practiced as an empirical rule.

Few reports exist regarding the treatment strategy for chylothorax following pulmonary resection. Hence, we report the clinical course and the validity of treatments for postoperative chylothorax occurring in our hospital.

## Methods

This study was approved by the Shizuoka Cancer Center Institutional Review Board (approval no. 56-2020-1-3), which waived the requirements for informed consent owing to the study’s retrospective nature.

Between September 2002 and March 2018, the 2019 patients who underwent lobectomy, bilobectomy, or pneumonectomy with mediastinal lymph node dissection for primary lung cancer at Shizuoka Cancer Center Hospital, Shizuoka, Japan were investigated. The diagnostic criteria for chylothorax were that the drainage of the pleural drain was evidently white and turbid, or the pleural effusion contained a triglyceride level of > 110 mg/dL. The medical records of these patients were reviewed for age, sex, surgical procedures, pathologic findings, amount of chest tube drainage, and treatment methods of the chylothorax. The pathological stage was determined according to the 7th edition of the Tumor (T) Node (N) Metastases (M) classification for lung cancer proposed by the International Association for the Study of Lung Cancer [5].

In this study, we retrospectively analyzed the clinical courses and the adequacy of treatments for postoperative chylothorax.

## Results

A total of 37 cases (20 men, 17 women; median age, 70 years; age range 33–80 years) with postoperative chylothorax were observed among 2019 patients (1.8%). Two patients underwent a bilobectomy and 35 patients underwent a lobectomy. Lobectomy on the right side was performed in 25 patients, including 20 upper, 3 lower, and 2 middle lobectomies. Lobectomy on the left side was performed in 10 patients, including 8 upper and 2 lower lobectomies. In addition, selective and systematic mediastinal lymph node dissections were performed in 17 and 20 cases, respectively. Right upper lobectomy was the most common; however, the same result was obtained when comparing the frequency in each resected lobe. The patient characteristics are summarized in Table 1. No patient received induction therapy. Complete video-assisted thoracoscopic surgery was performed in 1 patient (3%). The pathologic lymph node metastases were stage pN0 in 33 patients (89%), pN1 in 1 patient (3%), and pN2 in 3 patients (8%).

**Table 1:** Patient characteristics (n = 37)

Variables	
Age (years)	70 (33–80)
Sex (male/female)	20 (54%)/17 (46%)
Comorbidity^a^ (hypertension/diabetes/asthma/COPD/arrhythmia)	9 (24%)/4 (11%)/3 (8%)/2 (5%)/2 (5%)
Induction therapy (yes/no)	0 (0%)/37 (100%)
Lobectomy	35 (95%)
Right upper/middle/lower lobe	20 (3.0%^b^)/2 (2.0%^b^)/3 (0.8%^b^)
Left upper/lower lobe	8 (1.9%^b^)/2 (0.7%^b^)
Bilobectomy (right middle and lower lobes)	2 (5%)
Operation side (right/left)	27 (75%)/10 (25%)
Right mediastinal lymph node dissection (superior only/inferior only/both groups)	16 (43%)/0 (0%)/11 (30%)
Left mediastinal lymph node dissection (superior only/inferior only/both groups)	2 (5%)/2 (5%)/6 (16%)
Approach (c-VATS/Thoracotomy)	1 (3%)/36 (97%)
Pathologic N stage (N0/N1/N2)	33 (89%)/1 (3%)/3 (8%)
Pathologic stage (IA/IB/IIA/IIB/IIIA/IIIB)	18 (49%)/12 (32%)/3 (8%)/1 (3%)/3 (8%)

Data are presented as median (range) or number

*COPD* chronic obstructive pulmonary disease, *c-VATS* complete video-assisted thoracoscopic surgery

^a^Some patients had more than one comorbidity

^b^The frequency is shown for each resected lung lobe

When comparing the areas of the dissected lymph nodes, in all right-sided operations, superior mediastinal lymph node dissection was performed, and no chylothorax was noted in the group in which only the inferior mediastinal lymph node was dissected. In the left-sided operations, chylothorax occurred in the superior mediastinal lymph node dissection only, the inferior mediastinal lymph node dissection only, and both groups (Table 1).

All the patients were initially treated conservatively with a low-fat diet (fat < 10–30 g/day), prohibiting any consumption of fat-containing food or drink, and the amount of chylous drainage fluid was measured. Pleurodesis was performed by injecting a mixture of 10 Klinische Einheit units of OK-432 (a heat- and penicillin-treated lyophilized preparation of the SU strain of *Streptococcus pyogenes* A3; Picibanil; Chugai Pharmaceutical, Tokyo, Japan) and 100 mL isotonic saline into the thoracic cavity through a chest tube. Chest tube removal was considered when the chest tube drainage fluid was serous and the volume was at least < 200 mL per day.

Thirty-five patients (95%) were relieved with conservative treatment. No patient was treated with complete oral intake cessation and total parenteral nutrition. When the standard for drainage was set to 500 mL on the first day following the introduction of a low-fat diet, 28 cases were < 500 mL and 9 cases were ≥ 500 mL. Reoperation was performed in 2 of these 9 cases; and both cases showed a drainage volume ≥ 1000 mL per day. Leak points of chyle were revealed in front of the trachea at the lower end of the right superior mediastinum and root of the left common carotid artery at the upper edge of the aorta, respectively. The injuries were covered using a TachoComb fibrinogen tissue sealing sheet (CSL Behring, Tokyo, Japan). In both the cases, the thoracic duct was ligated at a level slightly above the diaphragm. After reoperation, the chest tube drainage decreased, and the chylothorax was treated at a median of 5 days (range 4–6) following reoperation and a median of 6.5 days (range 5–8) following the initial surgery.

**Fig. 1:**
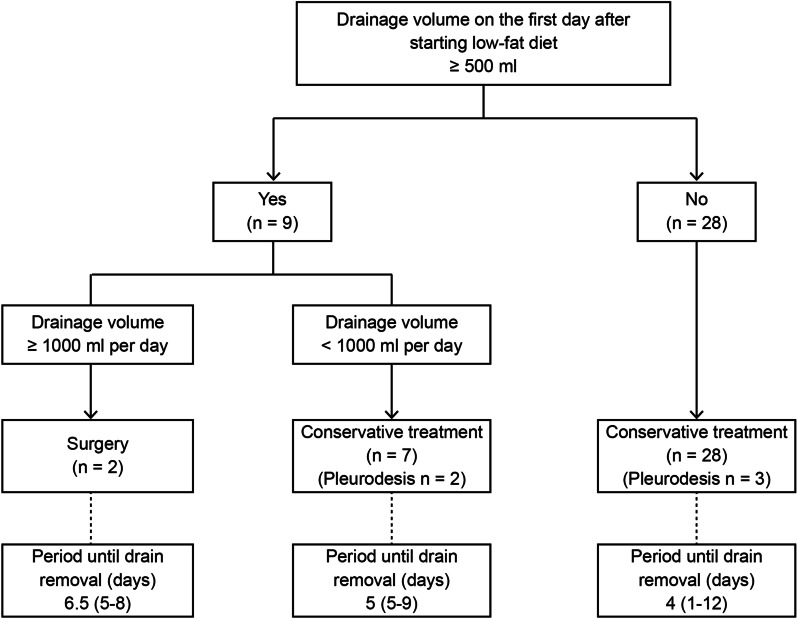
Treatment progression chart. The treatment progression when the first day drainage standard is set to 500 mL following the introduction of a low-fat diet. The period until drain removal is counted from the day when chylothorax is diagnosed. Data are presented as median (range)

In the other 7 cases, conservative treatment provided relief, based on the assumptions that the turbidity of the pleural effusion did not worsen over time, and the drainage volume decreased within 3 days. Pleurodesis was performed in 2 of the 7 cases. Chest tubes of these 7 cases were removed at a median of 5 days (range 5–9) following the initial surgery (Fig. 1). There was 1 case in which the daily drainage volume exceeded 500 mL on the third day, and it took 9 days until the chest drain could be removed (Table 2).

**Table 2:** Nine cases where drainage volume exceeds 500 mL first day after introduction of low-fat diet

Case	Treatment	Chest tube drainage (mL)			Period until drain removal (days)
		POD 1	POD 2	POD 3	
1	Surgery	1975	–	–	5
2	Conservative treatment	990	840	280	5
3	Conservative treatment	820	940	650	9
4	Surgery	605	1070	–	8
5	Conservative treatment	600	250	150	7
6	Conservative treatment	600	150	100	7
7	Conservative treatment	550	450	350	5
8	Conservative treatment	500	250	150	9
9	Conservative treatment	500	310	260	5

*POD* postoperative day

In addition, conservative treatment was successful in all 28 patients whose drainage did not exceed 500 mL on the first day, their chest tubes were removed at a median of 4 days (range 1–12) following the initial surgery (Fig. 1). Pleurodesis was performed in 3 cases.

In summary, conservative treatment was successful if the amount of drainage was < 500 mL on the first day after starting a low-fat diet. Furthermore, with the exception of cases with excessive drainage of ≥ 1000 mL in one day, when the drainage on the first day was ≥ 500 mL, the cases in which the drainage tended to decrease within 3 days recovered by conservative treatment.

Thirty-day death did not occur in any of the patients. Finally, 4 patients developed arrhythmia and 1 acute renal failure. All of these patients were treated conservatively.

## Discussion

Chylothorax, although relatively rare, is known as a serious complication and has a variety of causes. In thoracic surgery, chylothorax is caused by a thoracic duct injury due to mediastinal lymph node dissection, especially when performed for primary lung cancer. The incidence of postoperative chylothorax following lung resection is estimated to be 0.25–3% [3, 6] and is known to occur more often in the right thoracic cavity [7]. Previous studies have reported that lobectomy, right side surgery, robotic surgery, and pathological N2 disease are the most common causes of chylothorax [2]. In addition, Uchida et al. [8] stated that the most frequent chyle leak point was the #4R lymph node.

To date, there have been multiple discussions regarding the treatment methods for postoperative chylothorax [2–4, 9–11]. The principles of conservative treatment include efficient pleural drainage, sufficient residual lung expansion, and pleurodesis for reducing the dead space in the thoracic cavity. Furthermore, we attempted to close the fistula by reducing lymphatic flow in the thoracic duct using a low-fat diet, fasting, and total parenteral nutrition [12]. The first choice of treatment is conservative treatment, and it is reported that approximately 70–80% of the cases are relieved by conservative treatment alone [4].

Since chyle contains triglycerides, lymphocytes, proteins, and electrolytes, the loss of a large amount of chyle results in malnutrition and impaired immune function [4]. Therefore, for cases in which conservative treatment is ineffective, surgical treatments involving closure of the damaged site of the thoracic duct or ligation of the thoracic duct are considered. Regarding surgical indications, Shimizu et al. [4] argued that reoperation should be performed if drainage fluid ≥ 500 mL is obtained on the first day following fasting and total parenteral nutrition. In addition, Takuwa et al. [3] concluded that reoperation should be considered if there is drainage ≥ 500 mL per day after starting a low-fat diet (≤ 10 g).

In our study, with the exception of cases with excessive drainage of ≥ 1000 mL in one day, when the drainage on the first day following the introduction of a low-fat diet was ≥ 500 mL, the cases in which the drainage tended to decrease within 3 days could be cured conservatively. This suggests that even in cases in which the chyle leakage exceeds 500 mL daily, if a patient's general condition is maintained, the course could be observed conservatively at least until the third day. There was no noticeable difference in the duration of chest drainage between the surgery group and the conservative treatment group, and the maintenance period did not exceed 2 weeks even in the conservative treatment group. However, even if the drainage decreased by the third day, in the case where the drainage still exceeds 500 mL per day, the drainage period may be prolonged.

The present study has certain limitations. First, this study was a retrospective review that only included patients from a single institution. Second, the assessment regarding the properties of pleural effusion was subjective, and there was no objective index excluding the amount of drainage.

There was no clear protocol regarding the indication of pleurodesis in our hospital, and it was decided by the discretion of the attending physician. Although changes in the drainage amount did not suggest any indications for pleurodesis in this study, past reports have revealed that chylothorax could be controlled by pleurodesis using drugs such as OK-432 [13]. A sufficient therapeutic effect could be expected in cases in which the expansion of the residual lung is well attained and the dead space in the thoracic cavity is small despite the drainage volume being large. Therefore, since this is a less invasive treatment than reoperation, future research based on the accumulated cases is warranted.

## Conclusions

**Fig. 2:**
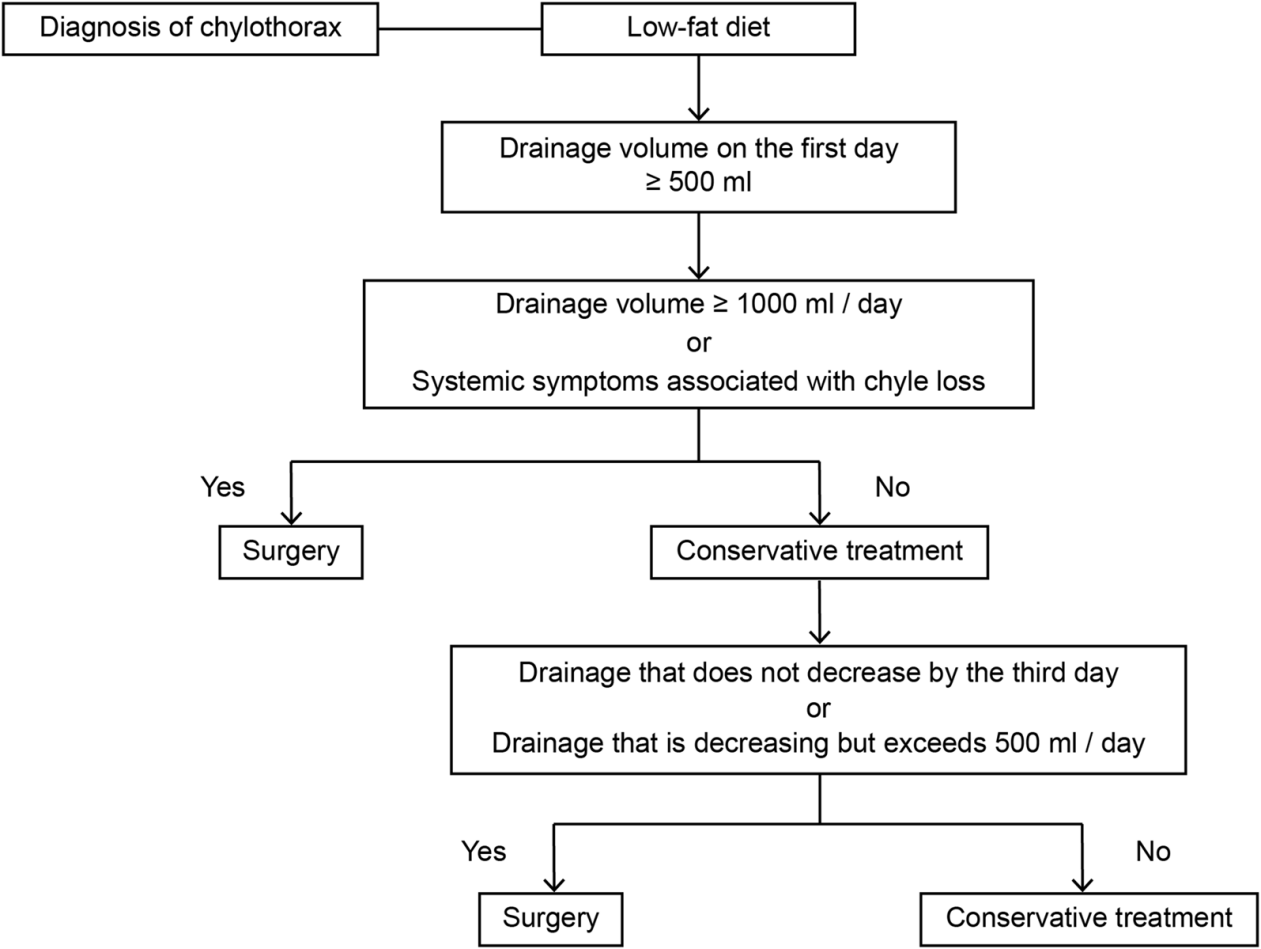
Treatment flow chart. Treatment flow chart for cases where the amount of drainage exceeds 500 mL on the first day following the introduction of a low-fat diet for postoperative chylothorax

Even when the daily drainage volume exceeds 500 mL following a low-fat diet, there were many cases that could be relieved by conservative treatment. The indication for reoperation needs to be carefully considered. We summarize the flow chart of treatment in cases where the amount of drainage exceeds 500 mL on the first day following the introduction of a low-fat diet for postoperative chylothorax according to the clinical courses and treatments in our hospital (Fig. 2). We consider surgery for cases in which the amount of drainage exceeds 1000 mL per day or where systemic symptoms associated with chyle loss are noted; in other cases, follow-up is possible. Furthermore, if by the third day there is no decrease in drainage, or if there is a decreasing tendency and the daily drainage still exceeds 500 mL, surgery is considered; otherwise, conservative treatment could be successful.

## Data Availability

The datasets generated and analyzed during the current study are not published due to the use of internal records of patient data and established privacy policies, but will be available from the corresponding author upon reasonable request.

## Abbreviation

TNM: Tumor node metastases
